# Successful Treatment of Necrotizing Fasciitis and Streptococcal Toxic Shock Syndrome with the Addition of Linezolid

**DOI:** 10.1155/2017/5720708

**Published:** 2017-02-19

**Authors:** Hana Rac, Karine D. Bojikian, Jose Lucar, Katie E. Barber

**Affiliations:** ^1^University of Mississippi Medical Center, 2500 North State Street, Jackson, MS, USA; ^2^University of Mississippi School of Pharmacy, 2500 North State Street, Jackson, MS, USA

## Abstract

Necrotizing fasciitis is a deep-seated subcutaneous tissue infection that is commonly associated with streptococcal toxic shock syndrome (TSS). Surgical debridement plus penicillin and clindamycin are the current standard of care. We report a case of necrotizing fasciitis and streptococcal TSS where linezolid was added after a failure to improve with standard therapy. Briefly after isolation of* Streptococcus pyogenes* from tissue cultures, the patient underwent two surgical debridement procedures and was changed to standard of care therapy. While the patient was hemodynamically stable, the patient's wounds, leukocytosis, and thrombocytopenia all progressively worsened. After initiation of linezolid, the patient slowly improved clinically. The present report is the first to highlight the role of linezolid in streptococcal necrotizing fasciitis and TSS not improving with standard therapy.

## 1. Introduction

Necrotizing fasciitis is a deep-seated subcutaneous tissue infection that can result in the destruction of both fascia and fat [1]. These infections are commonly associated with streptococcal toxic shock syndrome (TSS) and are often defined by the early onset of shock and organ failure and the isolation of group A* Streptococcus* (GAS) from the site of infection. Predisposing factors to necrotizing fasciitis due to* Streptococcus pyogenes* include burns, cuts, surgical procedures, childbirth, blunt trauma, and muscle strain.

Streptococcal pyogenic exotoxins are suspected to play a critical role in the pathogenesis of streptococcal TSS [1]. Treatment requires surgical debridement of necrotic tissue along with antimicrobials, typically including penicillin and clindamycin. The addition of clindamycin to penicillin for the treatment of streptococcal TSS is recommended as adjunctive therapy after immediate source control due to its toxin suppression activity [2]. While not commonly used for this indication, linezolid has also demonstrated in* in vitro* studies the ability to reduce* Streptococcus pyogenes* toxin synthesis or activity including protein M, streptolysin O, DNase, Spe B, Spe A, and F protein [3–5].

Here, we report a case of GAS necrotizing fasciitis and TSS in which the patient's outcome was influenced by the addition of linezolid to the antibiotic regimen.

## 2. Case Presentation

**Figure 1:**
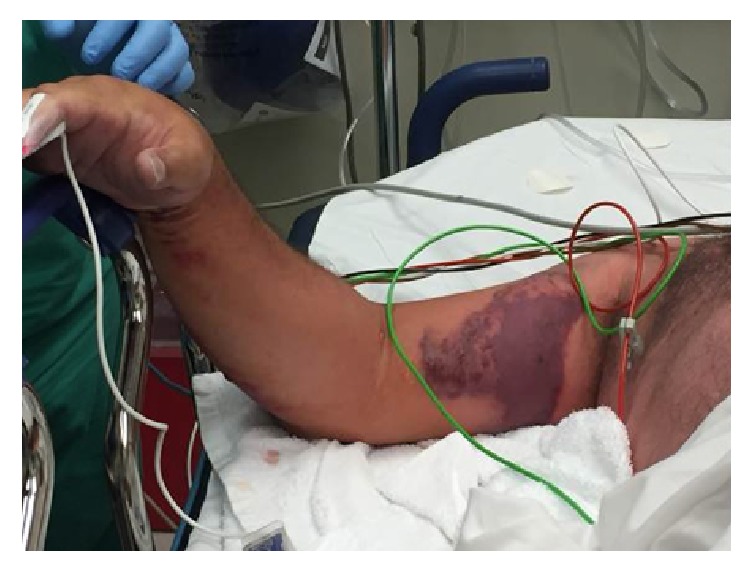
Right upper extremity on the day of presentation.

**Figure 2:**
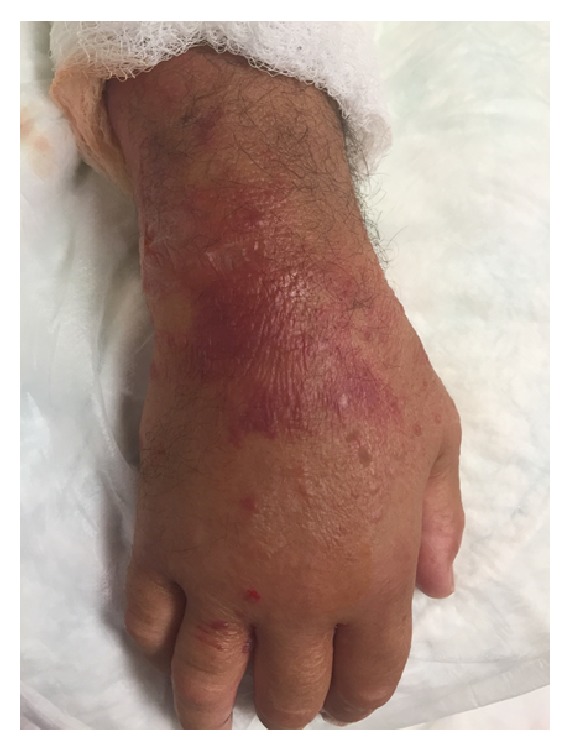
Right hand on the day of presentation.

**Figure 3:**
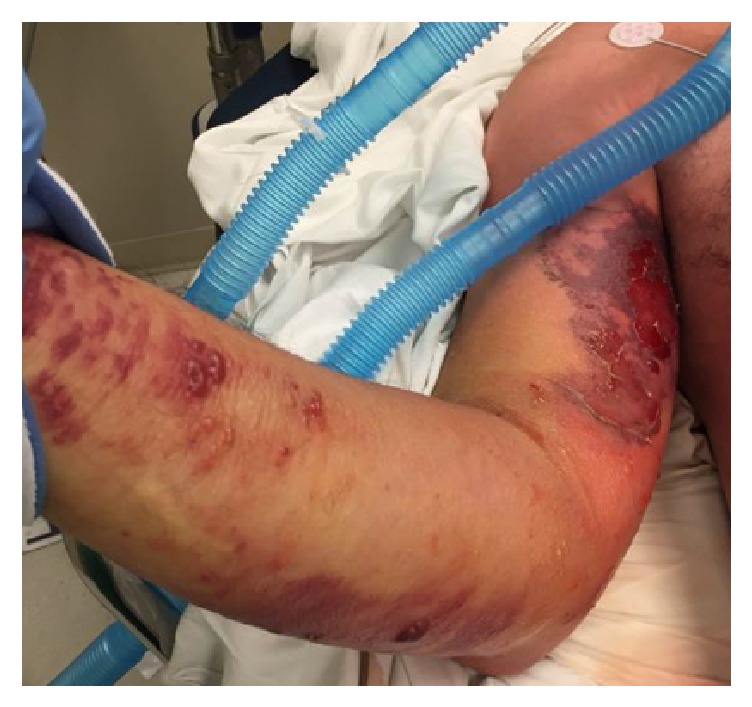
Right upper extremity progression prior to fasciotomy.

**Figure 4:**
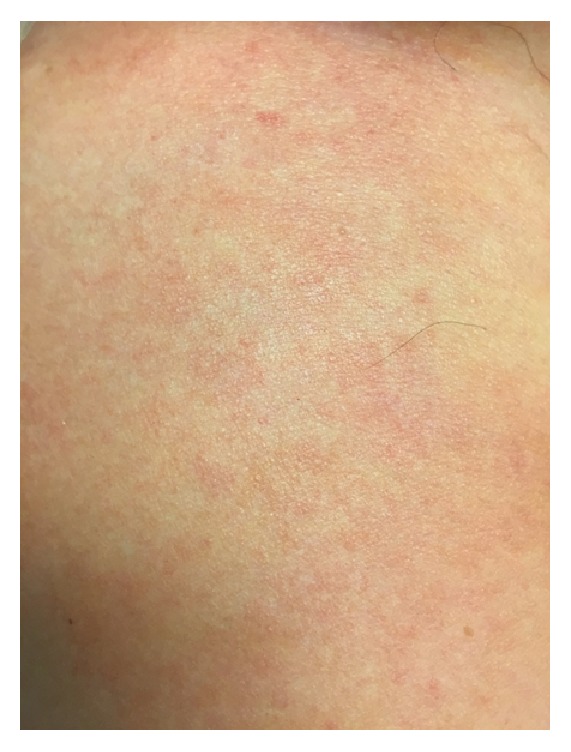
Maculopapular rash noted on admission on the patient's chest.

A 67-year-old Caucasian man with a history of coronary artery disease and sleep apnea presented to an outside hospital after reportedly suffering a bug bite on the right middle finger while traveling in the southeastern United States two days prior to admission. He presented with fever (39.4°C), a white blood cell (WBC) count of 2.9 th/cmm, serum creatinine (SCr) of 2.3 mg/dL, and blood pressure of 120/75 mmHg. After developing rapidly progressive right upper extremity (RUE) swelling, pain, and necrotic blisters, he was transferred to the University of Mississippi Medical Center via helicopter secondary to concerns of necrotizing fasciitis and sepsis. During transportation, his blood pressure dropped to 70/50 mmHg, and he required aggressive volume resuscitation. Upon arrival, the patient was noted to have swelling of the RUE with diffuse large blisters (Figures 1–3) in addition to a generalized maculopapular rash (Figure 4). He remained hypotensive (90/50 mmHg) and tachycardic (150 beats per minute) despite adequate fluid resuscitation, requiring vasoactive therapy with norepinephrine and vasopressin. He was subsequently intubated for hypoxia, respiratory failure, and altered mental status. Computerized Tomography of the RUE displayed extensive skin and soft tissue edema but no gas or abscesses. Blood cultures were obtained, and the patient was taken to the operating room (OR) for an emergent fasciotomy of the RUE and wound cultures. Two longitudinal incisions, one over the medial aspect of proximal RUE and the other one over the posterior aspect of the distal RUE, were made. Findings included edematous subcutaneous tissue and no air tracking along the fascia, and muscles appeared healthy. Damp to dry dressing was applied in both incisions. Right radial pulse was noted on Doppler at completion of case. Pertinent labs on admission were as follows: WBC, 2.2 th/cmm, platelets, 179 th/cmm, SCr, 2.68 mg/dL, creatine kinase (CK), 5704 units/L, pH, 7.21, PaO_2_, 138 mmHg, and PaCO_2_, 43 mm/Hg. On the day of admission, the patient was started on a regimen including vancomycin 15 mg/kg every 24 hours, clindamycin 600 mg every 8 hours, ceftazidime 1 g every 8 hours, and ciprofloxacin 400 mg every 12 hours.

On day 3, the patient clinically improved, no longer required vasopressors, and was extubated. Blood cultures remained negative, but tissue cultures demonstrating heavy growth of* Streptococcus pyogenes* were finalized on hospital day 4. Based upon these cultures, the patient's antibiotic regimen was deescalated to penicillin G potassium 4 million units every 4 hours and clindamycin 900 mg every 8 hours.

**Figure 5:**
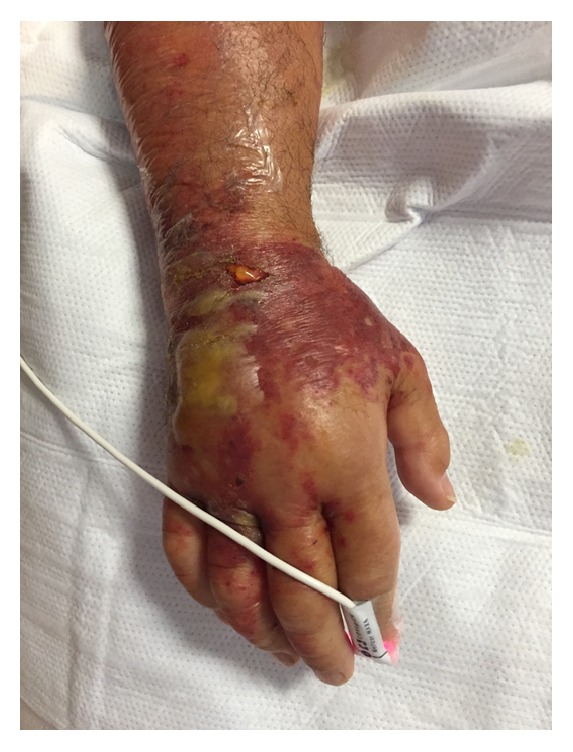
Worsening of skin and soft tissue changes on day 5 after initial improvement.

On day 5, the erythema and blisters on the RUE worsened (Figure 5). Additionally, WBC increased to 15.4 th/cmm and platelets dropped to 75 th/cmm. At this time, linezolid 600 mg IV every 12 hours was initiated for additional antistreptococcal activity and toxin suppression.

The patient returned to the OR on day 9 for partial wound closure. During the procedure, approximately 1 cm of skin subcutaneous tissue from the proximal RUE was excised due to necrosis leaving a healthy wound bed including exposed muscle and fascia. Negative pressure dressings were placed to open wound areas. Significant improvement in the swelling of the RUE was observed, and the laboratory values were within normal limits (WBC, 11.9 th/cmm; platelets, 259 th/cmm). On day 10, clindamycin therapy was discontinued due to apparent resolution of TSS. Linezolid and penicillin therapy were continued until day 13, at which time the patient was discharged solely on oral linezolid for the remaining course of therapy.

## 3. Discussion

We describe a case of RUE necrotizing fasciitis leading to the early onset of shock and organ failure with the isolation of GAS on culture, which is consistent with GAS TSS. The diagnosis of TSS was suspected early, which explains the empiric addition of clindamycin to the broad-spectrum regimen initially. While the patient's respiratory and hematologic status improved initially, the patient's wounds progressively worsened, the platelet count continued to drop, and WBC count increased. This led to the initiation of linezolid, an agent with both streptococcal bactericidal activity and antitoxin effects [6, 7]. Slowly, the patient began improving and was discharged on oral linezolid. While susceptibility testing for GAS was not done, clindamycin resistance is rare, and the initial improvement in the patient suggests that the isolate was not resistant [2].

Bacterial toxins are important mediators of septic shock due to their effects on the host immune system [8]. Beta-lactams lead to bacterial death due to disruption of bacterial cell walls, which then leads to the release of pathogen-associated molecular patterns that can induce a more robust immune response [9]. Agents that act on protein synthesis can suppress the synthesis of these toxins, which may decrease the host response. Linezolid and clindamycin both bind to the 50S subunit to inhibit protein synthesis [6, 10].* In vitro* data have shown that penicillin alone will increase endotoxins such as Spe A, while clindamycin and linezolid, alone or in combination, decreased Spe A production [5]. Other* in vitro* studies reveal a reduction in streptococcal endotoxins such as protein M, streptolysin O, DNase, Spe B, and F protein by linezolid [3, 4]. To our knowledge, the present work is the first to clinically suggest linezolid-induced suppression of TSS in addition to penicillin and clindamycin.

In summary, the patient was successfully treated for RUE necrotizing fasciitis and GAS TSS with a combination of penicillin, clindamycin, and linezolid. We conclude that the addition of linezolid in a patient not improving on penicillin and clindamycin contributed to a positive outcome in this case.
